# Regeneration and Plasticity Induced by Epidural Stimulation  in a Rodent Model of Spinal Cord Injury

**DOI:** 10.3390/ijms25169043

**Published:** 2024-08-21

**Authors:** Leonidas Gomes Angelin, Marcelo Nelson Páez Carreño, Jose Pinhata Otoch, Joyce Cristina Ferreira de Resende, Analía Arévalo, Lívia Clemente Motta-Teixeira, Marilia Cerqueira Leite Seelaender, Guilherme Lepski

**Affiliations:** 1 Laboratory of Medical Investigation, LIM26, Department of Experimental Surgery, Medical School, University of Sao Paulo, Sao Paulo 01246-903, Brazil; 2 Microelectronics and Materials Laboratory, Polytechnic School, University of Sao Paulo, Sao Paulo 05508-010, Brazil; 3 Laboratory of Neuroplasticity and Behaviour, Department of Physiological Sciences, Santa Casa de Sao Paulo School of Medical Sciences, Sao Paulo 01221-020, Brazil; livia.teixeira@fcmsantacasasp.edu.br

**Keywords:** spinal cord injury, motor recovery, epidural electric stimulation, neuroplasticity, regeneration

## Abstract

Traumatic spinal cord injury is a major cause of disability for which there are currently no fully effective treatments. Recent studies using epidural electrical stimulation have shown significant advances in motor rehabilitation, even when applied during chronic phases of the disease. The present study aimed to investigate the effectiveness of epidural electric stimulation in the motor recovery of rats with spinal cord injury. Furthermore, we aimed to elucidate the neurophysiological mechanisms underlying motor recovery. First, we improved upon the impact spinal cord injury model to cause severe and permanent motor deficits lasting up to 2 months. Next, we developed and tested an implantable epidural spinal cord stimulator device for rats containing an electrode and an implantable generator. Finally, we evaluated the efficacy of epidural electrical stimulation on motor recovery after spinal cord injury in Wistar rats. A total of 60 animals were divided into the following groups: (i) severe injury with epidural electrical stimulation (injury + stim, n = 15), (ii) severe injury without stimulation (group injury, n = 15), (iii) sham implantation without battery (sham, n = 15), and (iv) a control group, without surgical intervention (control, n = 15). All animals underwent weekly evaluations using the Basso, Beattie, Bresnahan (BBB) locomotor rating scale index, inclined plane, and OpenField test starting one week before the lesion and continuing for eight weeks. After this period, the animals were sacrificed and their spinal cords were explanted and prepared for histological analysis (hematoxylin–eosin) and immunohistochemistry for NeuN, β-III-tubulin, synaptophysin, and Caspase 3. Finally, NeuN-positive neuronal nuclei were quantified through stereology; fluorescence signal intensities for β-tubulin, synaptophyin, and Caspase 3 were quantified using an epifluorescence microscope. The injury + stim group showed significant improvement on the BBB scale compared with the injured group after the 5th week (*p* < 0.05). Stereological analysis showed a significantly higher average count of neural cells in the injury + stim group in relation to the injury group (1783 ± 2 vs. 897 ± 3, *p* < 0.001). Additionally, fluorescence signal intensity for synaptophysin was significantly higher in the injury + stim group in relation to the injury group (1294 ± 46 vs. 1198 ± 23, *p* < 0.01); no statistically significant difference was found in β-III-tubulin signal intensity. Finally, Caspase 3 signal intensity was significantly lower in the stim group (727 ± 123) compared with the injury group (1225 ± 87 *p* < 0.05), approaching levels observed in the sham and control groups. Our data suggest a regenerative and protective effect of epidural electrical stimulation in rats subjected to impact-induced traumatic spinal cord injury.

## 1. Introduction

Spinal cord injury represents a major problem worldwide, both for affected individuals and their loved ones, as well as the entire health care system. A global burden of disease study estimated as many as 20.6 million persons were affected by spinal cord injury in 2019, with an incidence of 0.9 million. This represents an age-standardized rate of 11.5 per 100,000 individuals and a global health impact of 6.5 million YLD (years of life with disability) [1,2].

Efforts to develop techniques that can promote spinal cord regeneration have come from several fronts, including the identification of neuroprotective pharmacological agents [3], stem cell transplantation [4,5], and neuro-modulation [6]. In the first line of research, animal studies and phase I/II trials have yielded promising agents. One of these is the sodium channel blocker Riluzole, which antagonizes the pre-synaptic release of glutamate and has been investigated in the treatment of acute cervical trauma. A recently published phase III trial with Riluzole did not reach the predetermined endpoint for efficacy in terms of the Upper Extremity Motor Score [7]. However, the trial did show some gains in functional recovery. Another initially promising agent, the tetracyclic antibiotic Minocycline was shown to provide neuroprotection in 25 patients enrolled in a phase II study [8] but no further report on the phase III trial followed. Similarly, a putatively beneficial effect of G-CSF (granulocyte colony-stimulating factor) in reducing lesion size has yet to be confirmed in clinical trials [9].

In another line of research, neural stem cells (NSCs) have been shown to differentiate into different neural cell types and promote some degree of tissue repair and pain control in animals [10,11]. Moreover, oligodendrocyte precursors have been investigated in phase I/II trials to test their potential to promote remyelination in the damaged cord [12]; however, no further evidence supporting this method has been reported. Another promising group of stem cells are mesenchymal stem cells (MSC) extracted from bone marrow, whose mechanism of action is thought to be mediated by local and systemic immunomodulation and trophic factor support. The results of recently published phase I/II trials using bone marrow [13], umbilical cord [14], or adipose tissue-derived MSC [15] reported improvements in ASIA grade, with some sensory gains observed, and one trial reported motor improvement [16].

This negative scenario changed drastically after initial reports of the potential benefits of epidural spinal cord stimulation (eSCS). In fact, eSCS has recently emerged as a promising approach to promote motor recovery in individuals with spinal cord injury (SCI). In animal studies, eSCS has been shown to induce neuroplasticity and facilitate functional improvements [6,17]. These findings have paved the way for clinical investigations in humans: several studies involving individuals with chronic complete SCI showed significant improvements in volitional motor control and quality of life after eSCS [18,19,20,21]. These studies highlight the potential of eSCS as a viable therapeutic strategy for SCI in humans. Although the electrophysiological changes in the damaged cord induced by epidural stimulation have been well clarified, especially in the animal model, possible cytoarchitectural or plastic changes induced by electrical stimulation remain poorly understood. Therefore, the main goal of the present study was to assess any changes in neuronal population, axo-dendritic sprouting, synaptic density, and apoptosis level that might explain any sensorimotor gain induced by eSCS in the animal impact model of spinal cord injury.

## 2. Results

### 2.1. BBB

**Figure 1:**
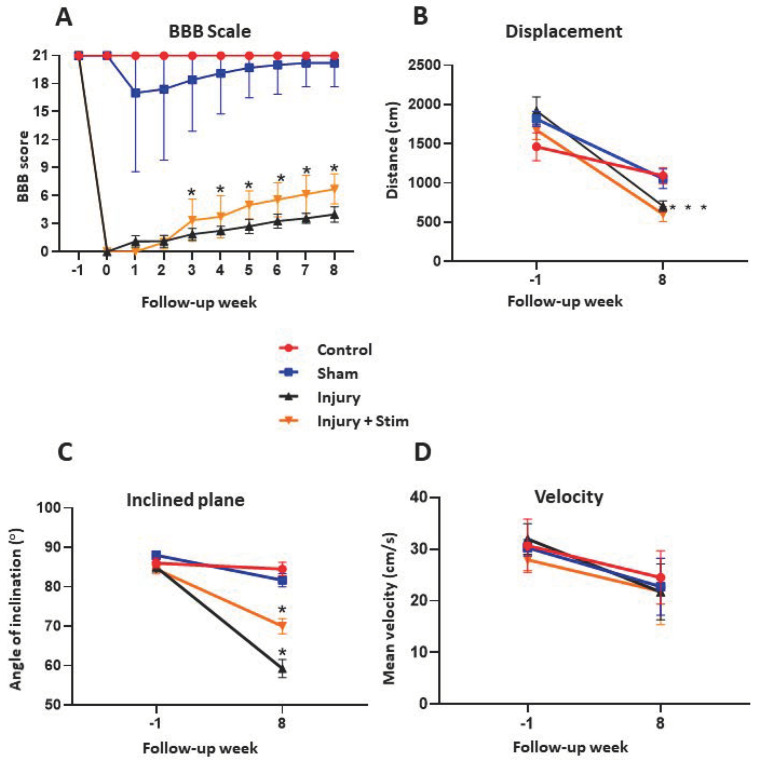
Behavioral tests after spinal cord injury and epidural stimulation. Four groups of animals were used for this purpose, 15 animals of each of the following: control, sham (stimulator implant but without battery), injury group (laminectomy and spinal cord injury through impact), and injury + stim. In (**A**), BBB score values were recorded weekly over 8 weeks of observation; notably, after the 5th week, a significant difference (*p* < 0.05) was observed between the injury + stim and injury groups, in favor of the stimulated group. In terms of general displacement in the open field test (**B**), both injured groups differed from controls but not from each other. In the inclined plane (**C**), there was a significant difference in performance in favor of the injury + stim group in relation to the injury group (*p* < 0.05). For velocity (**D**), no significant difference was observed among groups. * for *p* < 0.05, *** for *p* < 0.001.

In both groups of injured animals (with and without epidural stimulation), the BBB scale dropped to 0 immediately after the surgical procedure. Individuals in both groups recovered slowly and progressively until the 8th week. Notably, recovery in the group submitted to stimulation (injury + stim) was significantly more expressive than that observed in the injury group (non-stimulated) group (*p* < 0.05) from the 5th week until the end of the 8th week. However, the recovery peak did not reach pre-injury levels and remained at a maximum value of 7 in the injury + stim group (mean = 5 ± 6.05) and 5 in the injury group (mean = 4 ± 6.06). The control group did not show changes on the BBB scale and the sham group showed a small negative oscillation between the 1st and 3rd weeks; however, there was great variability (Figure 1A).

### 2.2. Inclined Plane

Relative to the control group, both injured groups displayed a significant decline in performance on the inclined plane test. However, in the injury + stim group, a tolerance to significantly higher inclination was noted (77° ± 7) in relation to the injury group (59° ± 2, *p* < 0.05) (Figure 1C).

### 2.3. Open Field

Relative to baseline values, all groups showed a reduction in the distance covered in the open-field behavioral test after the eight weeks of intervention. However, the largest reductions were observed in animals in the injury group (705 ± 220.2) and in the injury + stim (603 ± 316.6) group. Both groups showed a statistically significant difference in relation to the control group (*p* = 0.001) but no difference between each other, as shown in Figure 1B. Moreover, the animals’ displacement speed analysis revealed that all groups showed a decrease in velocity relative to preoperative values. However, no statistically significant differences were observed at any time, as shown in Figure 1D.

### 2.4. Histological Analysis

#### 2.4.1. Hematoxylin and Eosin

**Figure 2:**
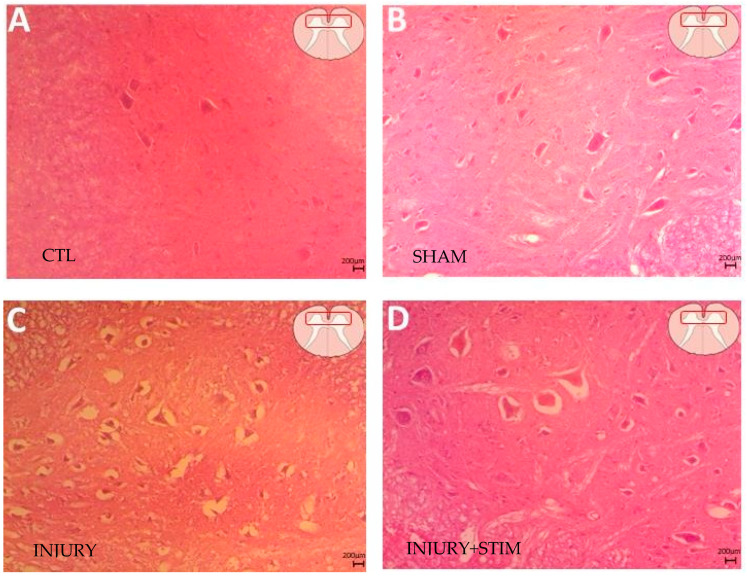
Microscope image of Hematoxylin and Eosin staining showing the (**A**) control, (**B**) sham, (**C**) injury, and (**D**) injury + stim groups. Note the (**C**) globular increase in cell bodies and pronounced cytoplasmatic vacuolization. In (**D**), changes in cellular cytoarchitecture were also observed, with regions showing vacuolization and globular enlargement of neuronal cell bodies, but these were less pronounced.

Histological analysis using hematoxylin and eosin staining enabled the identification of more significant tissue alterations in animals subjected to injury (Figure 2C), compared with the injury + stim group (Figure 2D). Evaluation of histological sections revealed that injured animals exhibited marked changes in neural morphology, including reduced cytoplasmic volume and more pronounced vacuolization. In contrast, no tissue damage was observed in animals from the control and sham groups (Figure 2A,B).

#### 2.4.2. Stereology for NeuN-Immunopositive Cells

**Figure 3:**
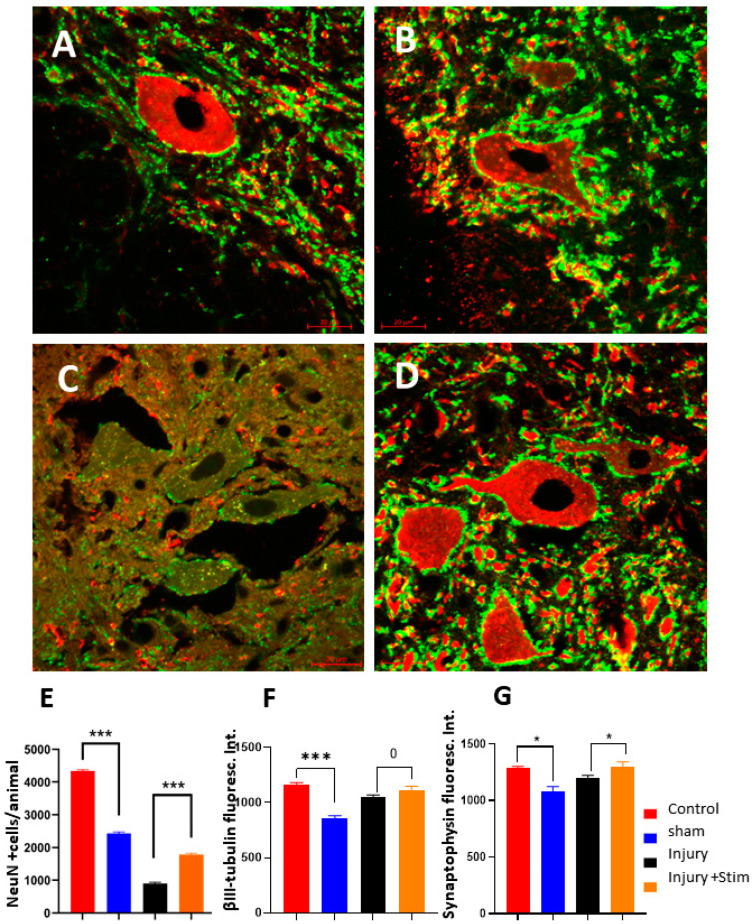
Cytoarchitectural changes induced by epidural stimulation. (**A**–**D**), confocal microscope pictures showing anti-synaptophysin revealed with AlexaFluor 488 (green) and anti-βIII-tubulin revealed with AlexaFLuor 594 (red), in a representative spinal cord axial slice in the control group (**A**), sham (**B**), injury group (**C**), and injury + stim (**D**) groups. All stainings were processed in parallel and all confocal pictures were taken with the same equipment settings. Note the intense cytoarchitectonic disorganization observed in (**C**), which is remarkably reversed under epidural stimulation (**D**) of all slices corresponding to the Z-height lesion epicenter −0.5 mm; all pictures were taken with the same confocal settings to allow for direct comparisons. In (**E**), estimative numbers of NeuN-positive neuronal nuclei, in terms of total cells per animal; note significantly larger numbers of NeuN+ cells in the injury + stim group compared with the injury group. In (**F**), βIII-tubulin fluorescence signal intensity was determined with an epifluorescence microscope (background fluorescence intensity subtracted from the total fluorescence in the frame, recorded in arbitrary units a.u.); no difference was seen between the injury + stim and injury groups. In (**G**), the same quantification was applied for synaptophysin signal intensity, which was significantly greater in the injury + stim group relative to the injury group (*p* < 0.05). 0 for no difference, * for *p* < 0.05, *** for *p* < 0.001.

In relation to NeuN-immunopositive cells, a significantly higher number of NeuN+ cells was counted in the group injury + stim, compared with the injury group (1783 ± 2 cells per animal in stim vs. of 897 ± 3 cells in the injury group, *p* < 0.001). Moreover, there was a reduced number of NeuN+ cells in the implanted animals (sham) compared with the controls (2433 ± 3 vs. 4334 ± 2, respectively, *p* < 0.001, Figure 3E.

#### 2.4.3. Synaptohysin and β-III-Tubulin Fluorescence

Confocal images revealed drastic disorganization in neuronal cytoarchitecture in the ventral horn of the damaged segments of the cord (see Figure 3C, rendering the recognition of βIII-tubulin or synaptophysin signals no longer possible) (for comparison, see Figure 3A, control group, and Figure 3B, sham groups). These alterations were partially reversed under epidural stimulation (Figure 3D). To allow direct comparison, pictures were taken from the same Z-axis coordinate (related to 0.5 mm above the lesion epicenter).

The analysis of fluorescence intensity for synaptophysin revealed a significantly higher signal in the injury + stim group (1294 ± 46 a.u.) relative to the injury group (1198 ± 23 a.u., *p* < 0.05). Furthermore, these values did not differ between the injury + stim and the control group (Figure 3G). Analogously for β-III-tubulin, significant differences were observed between the sham (859 ± 23 a.u.), injury (1049 ± 21 a.u.), and injury + stim (1116 ± 32 a.u.) groups relative to the control group (*p* < 0.001). However, no significant difference was observed between injury and injury + stim animals (1049 ± 21 vs 1116 ± 32 a.u., see Figure 3F).

#### 2.4.4. Caspase 3 Levels

**Figure 4:**
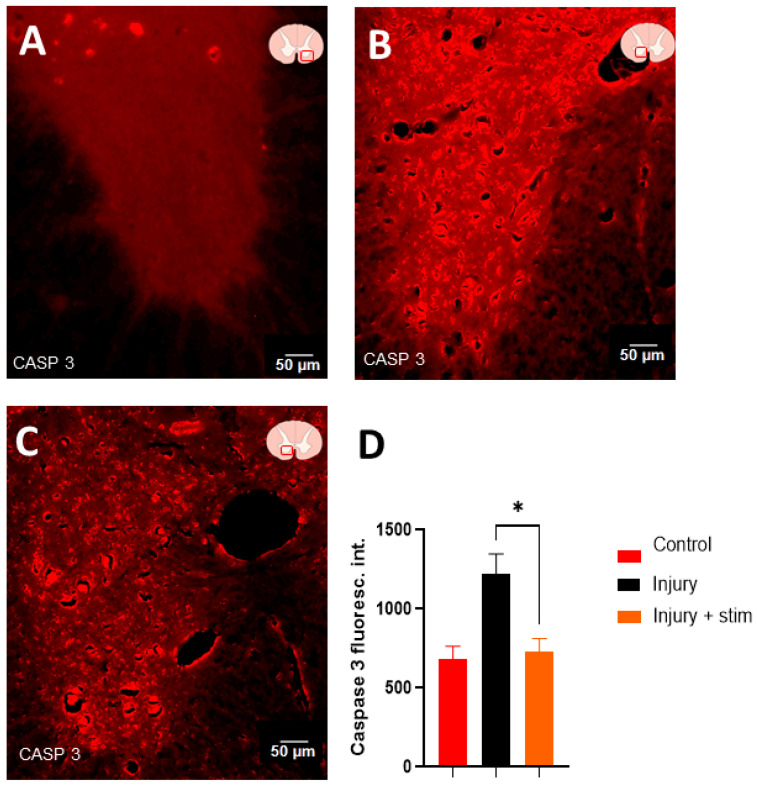
Representative photomicrographs of Caspase 3 immunostaining on transverse sections of the ventral horn in the spinal cord from control, injury, and injury + stim groups (revealed with Alexa 594). In (**A**), a representative image from the control group shows a relatively low expression level of Caspase 3. In (**B**), a representative image from the injury group with pronounced expression throughout the ventral horn is shown; note the morphological and structural changes in the tissue resulting from severe spinal cord injury. In (**C**), representative images from the injury + stim group exhibit notable immunofluorescence reactions, albeit with lower reactive luminescence compared with the injury group. Panel (**D**) presents the bar graphs of intensity measurements in terms of means and SEM; note the significantly different Caspase levels in the injury group, which are significantly higher when compared to the stim and control groups. Moreover, no difference in Caspase levels was seen between controls and stim. (* *p* < 0.05).

The fluorescence intensity analyses for Caspase 3 were conducted similarly to previous methods, revealing a statistically higher value observed in the injury group (1225 ± 87 a.u., illustrated in Figure 4B), in relation to the stim group (727 ± 123 a.u., *p* < 0.05, Figure 4C), and in the control group (681.9 ± 82, *p* < 0.05, Figure 4A). Moreover, the Caspase levels in the stim group approached the levels measured in controls and shams, as shown in Figure 4D.

## 3. Discussion

Preclinical research on the effects of epidural stimulation in spinal cord injury is critical. Growing evidence supports the clinical benefits of spinal cord stimulation (SCS) in humans with spinal cord injury, demonstrating significant motor improvements and functional gains [22,23]. However, the specific effects of SCS on spinal cord tissue remain largely unknown. Some studies have focused on the electrophysiological changes induced by SCS in animal models, using both computational simulations of neural networks and in vivo studies [6]. Despite these efforts, the cytoarchitectural and plastic changes induced by electric current in the spinal cord, as well as potential neuroprotective effects, remain largely unexplored.

Using an interesting mathematical model of neural computation and experimental verification in model rats, Moraud et al., in 2016, [6] sought to predict the firing potential of motor efferent fibers as a function of the exogenous current applied by epidural stimulation and the firing rate of afferents from neuromuscular spindles carried by fibers Ia and II. Previous studies showed that the spindle afferent model correlates with envelopes of bursts of electromyographic activity, which in turn correlate with the coupling of alpha-gamma motor activity [24,25]. It is believed that the motor response elicited in the hindlimbs to each pulse of epidural stimulation is preferentially mediated by thick myelinated fibers, particularly fibers involved in spindle-muscle sensory feedback [26,27,28]. Those muscle spindle afferents would then interact with supratentorial descending modulations to segmentally elaborate motor patterns [29]. Electrophysiological evidence dating back to Sherrington’s studies suggests that these regulatory circuits are common ancestors in the regulation of motor activity in vertebrates [30,31], are highly conserved in mammals [32], and may be recruited through epidural stimulation in humans [22,23].

In motor rehabilitation, the repetition of motor patterns (whether facilitated by a physiotherapist or exoskeletal prostheses) is known to induce spinal cord plasticity [33]. In this sense, epidural stimulation would act as one more (powerful) tool to provide these repetitions. The current interpretation of the effects of epidural stimulation is derived from mathematical simulation of the functioning of spinal cord-specific neural networks [26,34] and experimental studies [22,27]. Both lines of evidence suggest preferential activation of thick afferent fibers from neuromuscular spindles, which could recruit central-pattern generator (CPG) centers in the spinal cord [35,36]. This CPG activation would increase the excitability of spinal neurons themselves, integrating them into functioning sensory–motor networks [37]. Therefore, from an electrophysiological point of view, epidural stimulation modulates spindle afferent circuits in order to improve the motor program (such as changing gait speed). According to the most current models of understanding, epidural stimulation interacts with fusal sensory stimuli in two synergistic ways: (i) firstly, by stimulating fusal afferent fibers, thus favoring mono- and disynaptic excitatory efferents to medullary motoneurons and (ii) by strengthening the reciprocal inhibition of antagonists, thus favoring the alternating recruitment of agonists and antagonists necessary for proper gait [38]. According to this model, the interaction of these two mechanisms would provide the spinal cord with the ability to adjust and control the motor pattern independently of supratentorial centers [17,37]. An example of the complexity of medullary segmental patterns is what happens when the electric treadmill is stopped with the animal walking on it. At that moment, there is an increase in the activity of the extensor muscles and a decrease in the activity of the flexor muscles, promoting the transition from the gait state to static orthostasis [39]. This behavioral transition depends on the activation of mono- and disynaptic excitatory circuits within the spinal cord. Nevertheless, the modulatory inputs of CPG interneurons still need to be further clarified [40]. In our study, we were able to demonstrate objective motor improvement in the BBB score and in the inclined plane tests, which was significantly more pronounced in stimulated animals compared with injured ones. The time frame for this improvement (5 weeks) is very much in line with our complimentary observations on the cytoarchitectonic changes observed in the stimulated spinal cord. If the motor improvement was due to the electrophysiological recruitment of surviving cells and fibers through thick myelinated afferents, as mentioned initially, this should have been noticed immediately at the beginning of the stimulation. By contrast, our data suggest a more complex spinal cord reorganization being induced by external current.

Despite progress in understanding the electrophysiological changes caused by exogenous electrical current, the specific effects on the spinal cord’s neural structure remain largely unknown. Our study contributes to this understanding by demonstrating that stimulated animals exhibit an increase in neuronal populations and signals corresponding to synaptic structures. The growth in neural population may reflect increased neurogenesis induced by electrical stimulation, which is a recognized effect of electrical modulation [26,27]. Recently, Bang et al. (2024) administered direct electrical stimulation to the spinal cord injury site for 4 h daily, from 2 to 6 weeks following a T10 contusion SCI. At the conclusion of the study, spinal cord neuronal differentiation was assessed. Compared to animals that sustained SCI without stimulation, those receiving both SCI and stimulation exhibited a significantly higher number of neurons and nestin+ cells, indicating enhanced neuronal differentiation. Additionally, these animals showed improved tissue preservation and better locomotor recovery [41,42,43,44,45,46].

Evidence suggests that electrical spinal cord stimulation (eSCS) upregulates growth-promoting factors, enhancing axonal sprouting and facilitating the formation of new synapses. A key player in this process is Brain-Derived Neurotrophic Factor (BDNF), which promotes neuronal growth, neurogenesis, synaptic plasticity, and survival through multiple neuroprotective mechanisms, including anti-apoptosis, anti-oxidation, and autophagy suppression. It has been shown that increases in BDNF after SCI promote adaptive plasticity and functional recovery [47]. Additionally, pieces of evidence suggest that a combination strategy involving eSCS and adult tissue stem cell transplantation in an animal model of SCI improves both the structural repair and functional recovery of the injured spinal cord [48].

In addition to increased neurotrophin and neurogenesis, we propose that the observed increase in neuronal population is likely due to a decrease in late apoptosis following spinal cord injury. Studies have shown that in rats, expression levels of anti-apoptotic genes like Bcl2 and Bag1 can decrease threefold, one and three weeks post-injury [49]. Based on these findings, we speculate that stimulation inhibits the apoptosis in deafferented neuronal cells. Our data indicate that Caspase 3 signal intensity in stimulated animals approached levels observed in controls, which strongly supports this hypothesis. This suggests that stimulation may inhibit the apoptosis of deafferented neuronal cells, emphasizing the critical role of ongoing apoptosis in the pathophysiology of traumatic spinal cord injury.

Although advances have been made in understanding the electrophysiological changes induced by exogenous electrical current, very little is known about the effects of such stimulation on the medullary neural structure. In this study, we contributed to this knowledge by showing that stimulated animals display an increase in neuronal populations as well as the signal corresponding to synaptic structures. However, we know that the spinal cord is very poor in neurogenesis; indeed, neurogenesis does not occur in the adult animal’s spinal cord [4]. In our view, the most plausible hypothesis for this finding would be a decrease in late apoptosis after spinal cord injury, as mentioned above. Based on these findings, we speculate that stimulation inhibits the apoptosis of deafferented neuronal cells.

The second pathophysiological finding in stimulated animals was an increase in the synaptic fluorescence signal. It is well established that the exogenous current promotes synaptic maturation. The entry of ionic current into the cell activates several mechanisms of neuronal maturation through cAMP and calcium signaling, as well as activation of transcription factors like CREB, known to be involved in neurogenesis and synaptic maturation [50,51]. Very little is known about the structural changes induced by epidural stimulation. In humans with spinal cord injury (SCI) who have undergone spinal cord stimulation (SCS), plastic effects—such as gains in motor function even when stimulation is turned off—have been observed and documented in some clinical studies [22,34]. Explanations based solely on electrophysiological adjustments under external current are insufficient. The demonstration of neosynaptogenesis and reduced late apoptosis in neuronal cells, as reported here, sheds light on this issue by presenting anatomical reorganization that leads to the reintegration of motor networks in the damaged spinal cord, even in the absence of direct stimulation. The observed three-week timeframe for this plasticity to occur is consistent with the time required for the reorganization of motor networks due to the addition of new synapses.

Interestingly, the dendro-axonal sprouting, which could theoretically also be induced by exogenous current, was not observed with βIII-tubulin fluorescence measurements in our study. Notably, the βIII-tubulin fluorescence intensity in the sham group was significantly lower than in controls, unexpectedly. Given that βIII-tubulin stains highly selectively for neuronal cytoskeleton and the read-out of its signal intensity in the epifluorescent microscope is very precise, we assume that the sham group suffered some degree of lesion caused by the electrode implantation itself, which was not apparent in the behavioral tests. This would also explain the slight functional deterioration in the BBB scores observed in the sham group during the first weeks after laminectomy. The high variability suggests that some of the 15 animals in this group might have suffered some injury related to the laminectomy itself. The full recovery observed afterward did not preclude a reliable comparison among the groups at the end of the observational period.

In conclusion, our study brings important insights into how eSCS may induce functional recovery after SCI. The evidence presented here favors (i) increased plasticity in terms of synaptogenesis and (ii) enhanced neuronal population in the lesioned cord area, most probably due to (iii) the neuroprotective effect, supported by reduced secondary apoptosis, noted even in late phases after injury. To the best of our knowledge, these changes induced by eSCS have not been reported so far. A complete understanding of those mechanisms may contribute to further improvements in the neuromodulation strategies for motor recovery in humans.

## 4. Materials and Methods

### 4.1. Animals and Ethics

All experimental procedures adhered to the Ethical Principles of Animal Experimentation adopted by the Brazilian College of Animal Experimentation (COBEA), and were approved by the Animal Ethics Committee of the Medical School of the University of São Paulo (protocol 2013/2018). Sixty adult male Wistar were maintained in the vivarium of the Medical School of the University of São Paulo under environmentally controlled conditions: temperature at 22 ± 2 °C, a dark–light cycle of 12:12 h, and food and water ad libitum.

### 4.2. Experimental Design

**Figure 5:**
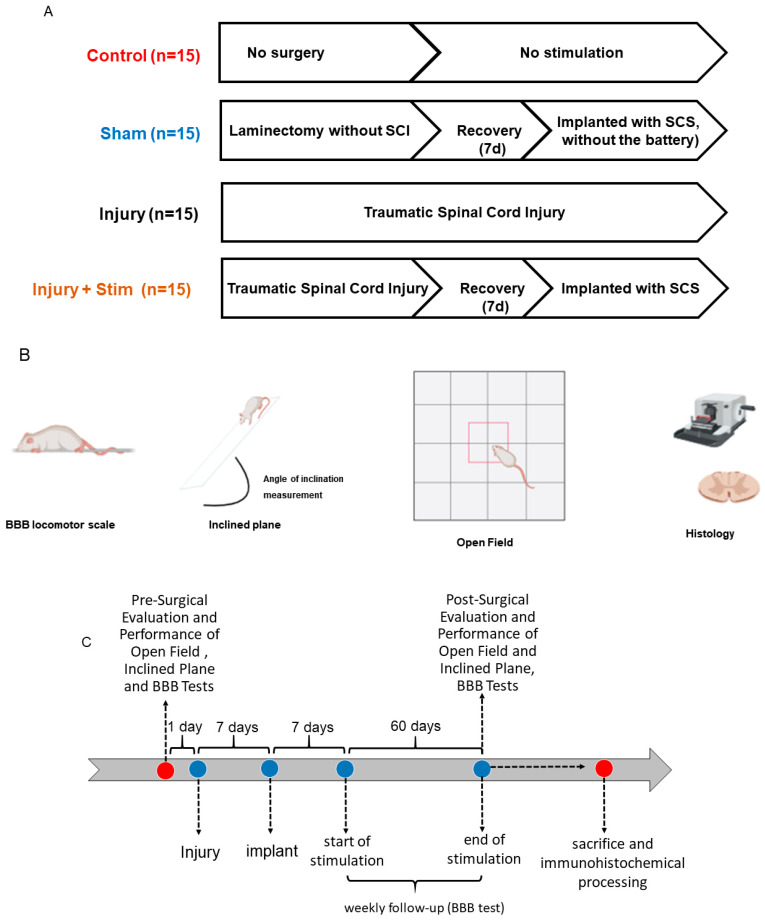
Schematic representation of the experiments. In (**A**), rats were divided into four groups: control group (without spinal cord injury-SCI, n = 15), sham group (implanted with spinal cord stimulation without the battery-SCS, n = 15), injury group (traumatic SCI, n = 15), and injury + stim group (submitted to traumatic SCI and full SCS, n = 15). Seven days post-SCI, the sham and stimulation groups had their spinal cords re-exposed. A paddle electrode and an internal pulse generator were implanted. The device delivered a 30 Hz, 500 μs, 110 mV pulse for 40 min daily over 60 days. In (**B**), locomotor function was evaluated weekly using the BBB scale, muscle strength was tested using the inclined plane test, and locomotor activity was assessed using the open field test. After the behavioral tasks, the rats were perfused and their spinal cords were processed for histological analysis. In panel (**C**), the timeline illustrates the sequence of experimental events, including pre-surgical evaluations, weekly analyses, final assessments, animal sacrifice, and immunohistochemical processing.

The experimental design of the present study is illustrated in Figure 5.

### 4.3. Impact Lesion Model

Thirty male Wistar rats weighing 250 to 300 g were subjected to intraperitoneal anesthesia with ketamine (100 mg/kg) and xylazine (10 mg/kg). After tricotomy and antisepsis of the surgical field, a median longitudinal skin incision was performed at levels T7–12; then, the paravertebral musculature was carefully separated from the vertebra laminae. Laminectomy was performed at levels T9 and T10, exposing the spinal cord and the intact dura mater. The SCI was applied using Benchmark Stereotaxic Impactor equipment (Impact One, Leica Biosystems, Richmond, IL, USA). Animals were fixed by the upper and lower vertebrae and a moderate injury was inflicted by dropping a weight of 2 mm in diameter at a velocity of 5 m/s, indentation depth in the cord programmed to be 2 mm, and lesion duration 0.1 ms. These lesion parameters were defined in a pilot study to ensure severe spinal cord injury with little spontaneous recovery over eight weeks of observation. During the surgical procedure, saline was replaced intraperitoneally to avoid hemodynamic instability.

At the end of the procedure, the muscle planes, subcutaneous tissue, and skin were sutured in multiple layers. Immediately post-operatively, animals were warmed under a bright light. They were housed in a temperature-controlled environment under a 12-h light–dark cycle with access to food and water ad libitum. All animals received subcutaneous injections of antibiotic (Flotril, 1 mg/kg) for 3 days and dipyrone (1 mg/kg) for 5 days; bladders were emptied twice daily until function returned. Supplemental Figure S1 illustrates the degree of tissue damage caused by this type of lesion in hematoxylin–eosin staining.

### 4.4. Spinal Cord Stimulation

**Figure 6:**
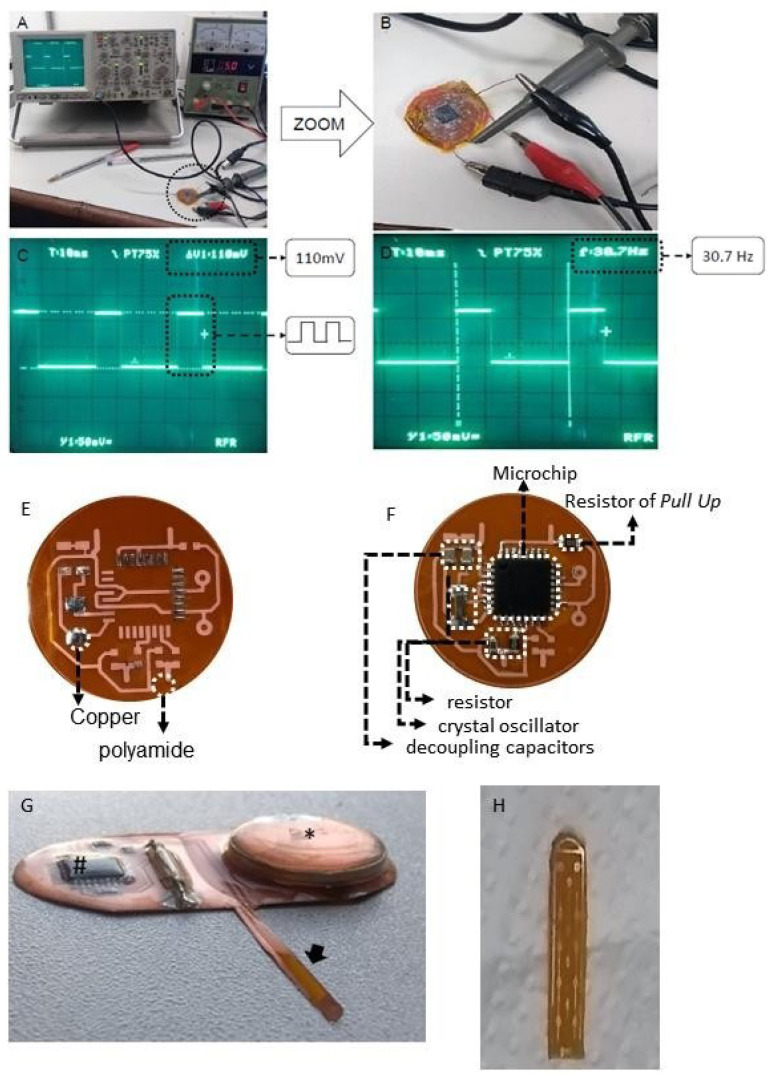
(**A**,**B**) bench test. Note the power supply adjusted to 5 V supplying the stimulation device; (**C**,**D**): oscilloscopic recording of the output current from the device (110 mV and 30.7 Hz). Figures (**E**,**F**) display the first version of the stimulator with its basic components, while Figure (**G**) depicts the final version of the device, # (Main microchip stimulation); * (Power supply); arrow (implantable electrode); (**H**) epidural implant electrode. The implantable pulse generator was designed and manufactured in our laboratory. It was conceived as a single piece, covered with dimethylpolysiloxane to avoid a battery leak. The lithium battery Sony CR2032, 220 mAh and 3 V, had enough charge to deliver a quadratic pulse of 30 Hz, 500 μs for 40 min a day over 60 days; the whole system required 1 mA/h. By the time of each animal’s sacrifice, the device was explanted and battery tension stability was assessed by millimeter (always higher than 2.9 V).

In our lab, we designed and manufactured an implantable miniaturized epidural stimulator (Figure 6). It was conceived as a single piece, covered with dimethylpolysiloxane to avoid battery leaks. The battery (lithium battery Sony CR2032, 220 mAh and 3 V; Sony, Tokyo, Japan) was potent enough to deliver a quadratic pulse of 30 Hz, 500 μs, 110 mV for 40 min a day over 60 days. Extensive electrical and duration testing, as well as biosecurity testing, were conducted in rats prior to the present study.

Seven days after SCI, 30 animals (15 from the sham and 15 from the injury + stim group) had their spinal cords re-exposed under general intraperitoneal anesthesia with ketamine (100 mg/kg) and xylazine (10 mg/kg). The paravertebral muscles were gently pulled away from the midline to expose the dura. The paddle electrode was then carefully inserted into the spinal canal in the caudal to cranial direction through the previously performed laminectomy. Finally, the integrated paddle electrode and pulse generator were implanted in a prepared subcutaneous pouch and the skin was sutured with mononylon 5-0. Immediately post-operatively, animals were warmed under a bright light. They were housed in a temperature-controlled environment under a 12-h light–dark cycle with access to food and water ad libitum. All animals received subcutaneous injections of antibiotic (Flotril, 1 mg/kg) for 3 days and dipyrone (1 mg/kg) for 5 days.

### 4.5. Behavioral Analysis

#### 4.5.1. Motricity/Basso, Beattie, Bresnahan (BBB) Locomotor Rating Scale

All behavioral analyses were performed one day before SCI and then weekly for eight weeks. Locomotor evaluations were conducted using the BBB scale (Basso et al., 1996), whose scores represent the sequential stages of recovery after SCI. The scale ranges from 0 to 21, where 0 represents no spontaneous movement of the hind paws and a score of 21 indicates normal locomotion [18].

#### 4.5.2. Inclined Plane

Muscle strength and endurance were evaluated using the inclined plane test. For this test, animals are placed face down on an adjustable inclined plane and the angle is increased from 0° to the point where the rat cannot maintain its position for 5 s. Two measurements were collected for each rat. These tests were conducted one day prior to the injury and one day before the animal’s sacrifice to avoid adaptation [19].

#### 4.5.3. Open Field

Overall locomotor activity was recorded using the Open Field device (Insight-06250-Insight^®^, Ribeirão Preto, Brazil), which tracks animals’ trajectory and provides information about velocity and walked distance. These tests were conducted one day prior to the injury and one day before the animal’s sacrifice to avoid adaptation [19].

### 4.6. Immunohistochemistry and Cell Quantification

Transcardial perfusion and immunohistochemistry. After eight weeks, the animals were euthanized by intraperitoneal lethal injection of ketamine (200 mg/kg), followed by transcardiac perfusion. The stimulation device was explanted and the tension in the battery was measured with a multimeter (>2.9 V indicated proper functioning). Their spinal cords were extracted and a 2 cm segment, including the entire area of the lesion (1 cm above and below the lesion epicenter), was frozen using isopentane and dry ice. Serial 60 μm sections were obtained from all spinal cord segments using a cryostat (CM3000, Leica, Nussloch, Germany).

The staining procedure was performed on glass slides, as previously reported [20,21]. In brief, the sections were rinsed 5 × 5 min with Phosphate-Buffered Saline (PBS) supplemented with Tween 0.05% and incubated in a blocking solution containing 5% goat serum and 0.3% Triton X-100 diluted in PBS for one hour at room temperature and then incubated with the primary antibodies anti-neuronal nuclei (NeuN, monoclonal, mouse, 1:1000, Millipore, Temecula, CA, USA, cat#MAB377), anti-βIII-tubulin (monoclonal, mouse, 1:1000, Millipore, Temecula, CA, USA, cat#AB9354), anti-synapto physin (monoclonal, mouse, 1:1000, Millipore, Temecula, CA, USA, cat#MAB5258-I), and anti-Caspase 3 (rabbit, 1:1000, Cell Signalling, cat#9665, Danvers, MA, USA) diluted in blocking solution and maintained at 4 °C for 24 h. After this period, the sections were rinsed again in PBS-Tween 5 × 5 min and incubated for an additional 2 h at room temperature with anti-mouse and anti-rabbit Alexa Fluor 488 and Alexa Fluor 594 (polyclonal, goat, 1:150, Molecular Probes, Eugene, OR, USA) diluted in 0.3% TritonX-100 in PBS. After another series of washes, the coverslips were mounted on slides using a fluorescent mounting medium (Dako, Glostrup, Denmark) to preserve immunofluorescence.

#### 4.6.1. Estimates of NeuN-Immunopositive Cells

The 2 cm cord segment comprising the lesion epicenter was scanned to acquire Immunofluorescence pictures digitalized at 2048 × 2028 megapixels, in 6450 × 6450 μm frames; 450 frames were quantified for each group. The absolute number of NeuN-immunopositive cells was determined semi-automatically using a Zeiss Axio Imager A2 microscope (Carl Zeiss, Gottingen, Germany) equipped with Zen blue software, version Zen 2 Blue (Carl Zeiss). The estimated total number of positive NeuN cells was calculated using Abercrombie’s correction formulas as follows, P = M/(D + M)A × N, where P = total cell number, M = section thickness, D = average diameter of the positive cells, A = number of counted cells, and N = number of cut series [52].

#### 4.6.2. Fluorescence Quantifications

Specifically for fluorescence measures of βIII-tubulin and Synaptophysin, we systematically selected 300 images from each group, in a sequential manner, in the equatorial line of the coverslip, from left to right, starting from rostral to caudal. Two regions of interest were selected in each picture (ROI’s), with one representing the total visual field (ROIt) and the other an area of background embracing 10% of the lowest signal intensity (ROIb). Mean fluorescence intensity (MFI) was calculated by subtracting MFIROIb from MFIROIt [50]. For Capase-3 fluorescence signal intensity measures, the same procedure has been adopted in 150 images in the control, injury group, and injury + stim groups.

### 4.7. Statistical Analysis

Statistical analyses were performed using JMP v.16.0 (SAS Institute, Cary, NC, USA) and graphs were built with GraphPad Prism, version 9.5.1 (GraphPad software, Inc., San Diego, CA, USA). We first tested normal distribution with the Shapiro–Wilk test. Normal data were compared by means of Student’s *t*-tests and ANOVAs, whereas nonparametric data were compared with the Mann–Whitney U test and Kruskal–Wallis test followed by Dunn’s post hoc test to compare among three or more samples. Significance was set at *p* equal to or less than 0.05. Summary statistics are presented in terms of means and standard errors.

## Data Availability

The original contributions presented in the study are included in the article and Supplementary Materials, further inquiries can be directed to the corresponding author.

## Supplementary Materials

The following supporting information can be downloaded at: https://www.mdpi.com/article/10.3390/ijms25169043/s1.

## References

[1] GBD Collaborator Network (2024). Global, regional, and national burden of disorders affecting the nervous system, 1990-2021: A systematic analysis for the Global Burden of Disease Study 2021. Lancet Neurol..

[2] GBD Collaborator Network (2023). Global, regional, and national burden of spinal cord injury, 1990–2019: A systematic analysis for the Global Burden of Disease Study 2019. Lancet Neurol..

[3] Hachem L.D., Ahuja C.S., Fehlings M.G. (2017). Assessment and management of acute spinal cord injury: From point of injury to rehabilitation. J. Spinal Cord. Med..

[4] Mariano E.D., Batista C.M., Barbosa B.J., Marie S.K., Teixeira M.J., Morgalla M., Tatagiba M., Li J., Lepski G. (2014). Current perspectives in stem cell therapy for spinal cord repair in humans: A review of work from the past 10 years. Arq. Neuropsiquiatr..

[5] Li J., Lepski G. (2013). Cell transplantation for spinal cord injury: A systematic review. Biomed. Res. Int..

[6] Moraud E.M., Capogrosso M., Formento E., Wenger N., DiGiovanna J., Courtine G., Micera S. (2016). Mechanisms Underlying the Neuromodulation of Spinal Circuits for Correcting Gait and Balance Deficits after Spinal Cord Injury. Neuron.

[7] Fehlings M.G., Moghaddamjou A., Harrop J.S., Stanford R., Ball J., Aarabi B., Freeman B.J.C., Arnold P.M., Guest J.D., Kurpad S.N. (2023). Safety and Efficacy of Riluzole in Acute Spinal Cord Injury Study (RISCIS): A Multi-Center, Randomized, Placebo-Controlled, Double-Blinded Trial. J. Neurotrauma.

[8] Casha S., Zygun D., McGowan M.D., Bains I., Yong V.W., Hurlbert R.J. (2012). Results of a phase II placebo-controlled randomized trial of minocycline in acute spinal cord injury. Brain.

[9] Takahashi H., Yamazaki M., Okawa A., Sakuma T., Kato K., Hashimoto M., Hayashi K., Furuya T., Fujiyoshi T., Kawabe J. (2012). Neuroprotective therapy using granulocyte colony-stimulating factor for acute spinal cord injury: A phase I/IIa clinical trial. Eur. Spine J..

[10] Lepski G., Batista C.M., Mariano E.D., Dale C.S., Cristante A.F., Britto L.R., Otoch J.P., Teixeira M.J., Morgalla M. (2019). Pain inhibition through transplantation of fetal neuronal progenitors into the injured spinal cord in rats. Neural Regen. Res..

[11] Lepski G. (2020). Transplantation of GABAergic precursors into the spinal cord to alleviate neuropathic pain. Ann. Palliat. Med..

[12] Fessler R.G., Ehsanian R., Liu C.Y., Steinberg G.K., Jones L., Lebkowski J.S., Wirth E.D., McKenna S.L. (2022). A phase 1/2a dose-escalation study of oligodendrocyte progenitor cells in individuals with subacute cervical spinal cord injury. J. Neurosurg. Spine.

[13] Saini R., Pahwa B., Agrawal D., Singh P.K., Gujjar H., Mishra S., Jagdevan A., Misra M. (2022). Efficacy and outcome of bone marrow derived stem cells transplanted via intramedullary route in acute complete spinal cord injury—A randomized placebo controlled trial. J. Clin. Neurosci..

[14] Albu S., Kumru H., Coll R., Vives J., Vallés M., Benito-Penalva J., Rodríguez L., Codinach M., Hernández J., Navarro X. (2021). Clinical effects of intrathecal administration of expanded Wharton jelly mesenchymal stromal cells in patients with chronic complete spinal cord injury: A randomized controlled study. Cytotherapy.

[15] Bydon M., Qu W., Moinuddin F.M., Hunt C.L., Garlanger K.L., Reeves R.K., Windebank A.J., Zhao K.D., Jarrah R., Trammell B.C. (2024). Intrathecal delivery of adipose-derived mesenchymal stem cells in traumatic spinal cord injury: Phase I trial. Nat. Commun..

[16] Morita T., Sasaki M., Kataoka-Sasaki Y., Nakazaki M., Nagahama H., Oka S., Oshigiri T., Takebayashi T., Yamashita T., Kocsis J.D. (2016). Intravenous infusion of mesenchymal stem cells promotes functional recovery in a model of chronic spinal cord injury. Neuroscience.

[17] Courtine G., Gerasimenko Y., van den Brand R., Yew A., Musienko P., Zhong H., Song B., Ao Y., Ichiyama R.M., Lavrov I. (2009). Transformation of nonfunctional spinal circuits into functional states after the loss of brain input. Nat. Neurosci..

[18] Basso D.M., Beattie M.S., Bresnahan J.C., Anderson D.K., Faden A.I., Gruner J.A., Holford T., Hsu C., Noble L., Nockels R. (1996). MASCIS evaluation of open field locomotor scores: Effects of experience and teamwork on reliability. Multicenter Animal Spinal Cord Injury Study. J. Neurotrauma.

[19] Rivlin A.S., Tator C.H. (1977). Objective clinical assessment of motor function after experimental spinal cord injury in the rat. J. Neurosurg..

[20] Lepski G., Jannes C.E., Wessolleck J., Kobayashi E., Nikkhah G. (2011). Equivalent neurogenic potential of wild-type and GFP-labeled fetal-derived neural progenitor cells before and after transplantation into the rodent hippocampus. Transplantation.

[21] Lepski G., Jannes C.E., Strauss B., Marie S.K., Nikkhah G. (2010). Survival and neuronal differentiation of mesenchymal stem cells transplanted into the rodent brain are dependent upon microenvironment. Tissue Eng. Part A.

[22] Hofstoetter U.S., Danner S.M., Freundl B., Binder H., Mayr W., Rattay F., Minassian K. (2015). Periodic modulation of repetitively elicited monosynaptic reflexes of the human lumbosacral spinal cord. J. Neurophysiol..

[23] Sayenko D.G., Angeli C., Harkema S.J., Edgerton V.R., Gerasimenko Y.P. (2014). Neuromodulation of evoked muscle potentials induced by epidural spinal-cord stimulation in paralyzed individuals. J. Neurophysiol..

[24] Prochazka A., Gorassini M. (1998). Ensemble firing of muscle afferents recorded during normal locomotion in cats. J. Physiol..

[25] Prochazka A., Gorassini M. (1998). Models of ensemble firing of muscle spindle afferents recorded during normal locomotion in cats. J. Physiol..

[26] Capogrosso M., Wenger N., Raspopovic S., Musienko P., Beauparlant J., Bassi Luciani L., Courtine G., Micera S. (2013). A computational model for epidural electrical stimulation of spinal sensorimotor circuits. J. Neurosci..

[27] Gerasimenko Y.P., Lavrov I.A., Courtine G., Ichiyama R.M., Dy C.J., Zhong H., Roy R.R., Edgerton V.R. (2006). Spinal cord reflexes induced by epidural spinal cord stimulation in normal awake rats. J. Neurosci. Methods.

[28] Wenger N., Moraud E.M., Raspopovic S., Bonizzato M., DiGiovanna J., Musienko P., Morari M., Micera S., Courtine G. (2014). Closed-loop neuromodulation of spinal sensorimotor circuits controls refined locomotion after complete spinal cord injury. Sci. Transl. Med..

[29] Levine A.J., Hinckley C.A., Hilde K.L., Driscoll S.P., Poon T.H., Montgomery J.M., Pfaff S.L. (2014). Identification of a cellular node for motor control pathways. Nat. Neurosci..

[30] Clarac F., Cattaert D., Le Ray D. (2000). Central control components of a “simple” stretch reflex. Trends Neurosci..

[31] Sherrington C.S. (1910). Flexion-reflex of the limb, crossed extension-reflex, and reflex stepping and standing. J. Physiol..

[32] Pierrot-Deseilligny E. (1989). Peripheral and descending control of neurones mediating non-monosynaptic Ia excitation to motoneurones: A presumed propriospinal system in man. Prog. Brain Res..

[33] Borton D., Micera S., Millán Jdel R., Courtine G. (2013). Personalized neuroprosthetics. Sci. Transl. Med..

[34] Rattay F., Minassian K., Dimitrijevic M.R. (2000). Epidural electrical stimulation of posterior structures of the human lumbosacral cord: 2. quantitative analysis by computer modeling. Spinal Cord..

[35] Angeli C.A., Edgerton V.R., Gerasimenko Y.P., Harkema S.J. (2014). Altering spinal cord excitability enables voluntary movements after chronic complete paralysis in humans. Brain.

[36] Danner S.M., Hofstoetter U.S., Freundl B., Binder H., Mayr W., Rattay F., Minassian K. (2015). Human spinal locomotor control is based on flexibly organized burst generators. Brain.

[37] Edgerton V.R., Courtine G., Gerasimenko Y.P., Lavrov I., Ichiyama R.M., Fong A.J., Cai L.L., Otoshi C.K., Tillakaratne N.J., Burdick J.W. (2008). Training locomotor networks. Brain Res. Rev..

[38] Talpalar A.E., Endo T., Löw P., Borgius L., Hägglund M., Dougherty K.J., Ryge J., Hnasko T.S., Kiehn O. (2011). Identification of minimal neuronal networks involved in flexor-extensor alternation in the mammalian spinal cord. Neuron.

[39] Quevedo J., Fedirchuk B., Gosgnach S., McCrea D.A. (2000). Group I disynaptic excitation of cat hindlimb flexor and bifunctional motoneurones during fictive locomotion. J. Physiol..

[40] Rybak I.A., Stecina K., Shevtsova N.A., McCrea D.A. (2006). Modelling spinal circuitry involved in locomotor pattern generation: Insights from the effects of afferent stimulation. J. Physiol..

[41] Bang W.S., Han I., Mun S.A., Hwang J.M., Noh S.H., Son W., Cho D.-C., Kim B.-J., Kim C.H., Choi H. (2024). Electrical stimulation promotes functional recovery after spinal cord injury by activating endogenous spinal cord-derived neural stem/progenitor cell: An in vitro and in vivo study. Spine J..

[42] Liu Q., Telezhkin V., Jiang W., Gu Y., Wang Y., Hong W., Tian W., Yarova P., Zhang G., Lee S.M.-Y. (2023). Electric field stimulation boosts neuronal differentiation of neural stem cells for spinal cord injury treatment via PI3K/Akt/GSK-3β/β-catenin activation. Cell Biosci..

[43] Becker D., Gary D., Rosenzweig E., Grill W., McDonald J. (2010). Functional electrical stimulation helps replenish progenitor cells in the injured spinal cord of adult rats. Exp. Neurol..

[44] Rodríguez-Barrera R., Rivas-González M., García-Sánchez J., Mojica-Torres D., Ibarra A. (2021). Neurogenesis after spinal cord injury: State of the art. Cells.

[45] Havelikova K., Smejkalova B., Jendelova P. (2022). Neurogenesis as a tool for spinal cord injury. Int. J. Mol. Sci..

[46] Rodríguez-Barrera R., Flores-Romero A., García E., Fernández-Presas A.M., Incontri-Abraham D., Navarro-Torres L., García-Sánchez J., Whaley J.J.J., Madrazo I., Ibarra A. (2020). Immunization with neural-derived peptides increases neurogenesis in rats with chronic spinal cord injury. CNS Neurosci. Ther..

[47] Dorrian R.M., Berryman C.F., Lauto A., Leonard A.V. (2023). Electrical stimulation for the treatment of spinal cord injuries: A review of the cellular and molecular mechanisms that drive functional improvements. Front. Cell. Neurosci..

[48] Zeng Y.S., Ding Y., Xu H.Y., Zeng X., Lai B.Q., Li G., Ma Y.H. (2022). Electro-acupuncture and its combination with adult stem cell transplantation for spinal cord injury treatment: A summary of current laboratory findings and a review of literature. CNS Neurosci. Ther..

[49] Yip P.K., Malaspina A. (2012). Spinal cord trauma and the molecular point of no return. Mol. Neurodegener..

[50] Lepski G., Jannes C.E., Nikkhah G., Bischofberger J. (2013). cAMP promotes the differentiation of neural progenitor cells in vitro via modulation of voltage-gated calcium channels. Front. Cell Neurosci..

[51] Schmidt-Hieber C., Jonas P., Bischofberger J. (2004). Enhanced synaptic plasticity in newly generated granule cells of the adult hippocampus. Nature.

[52] Furlanetti L.L., Cordeiro J.G., Cordeiro K.K., Garcia J.A., Winkler C., Lepski G.A., Coenen V.A., Nikkhah G., Döbrössy M.D. (2015). Continuous High-Frequency Stimulation of the Subthalamic Nucleus Improves Cell Survival and Functional Recovery Following Dopaminergic Cell Transplantation in Rodents. Neurorehabil. Neural Repair..

