# Supplementary figures and images for: Regeneration and Plasticity Induced by Epidural Stimulation in a Rodent Model of Spinal Cord Injury
Source: Int J Mol Sci. 2024 Aug 21;25(16):9043. doi: 10.3390/ijms25169043 (PMC11354918; doi:10.3390/ijms25169043)

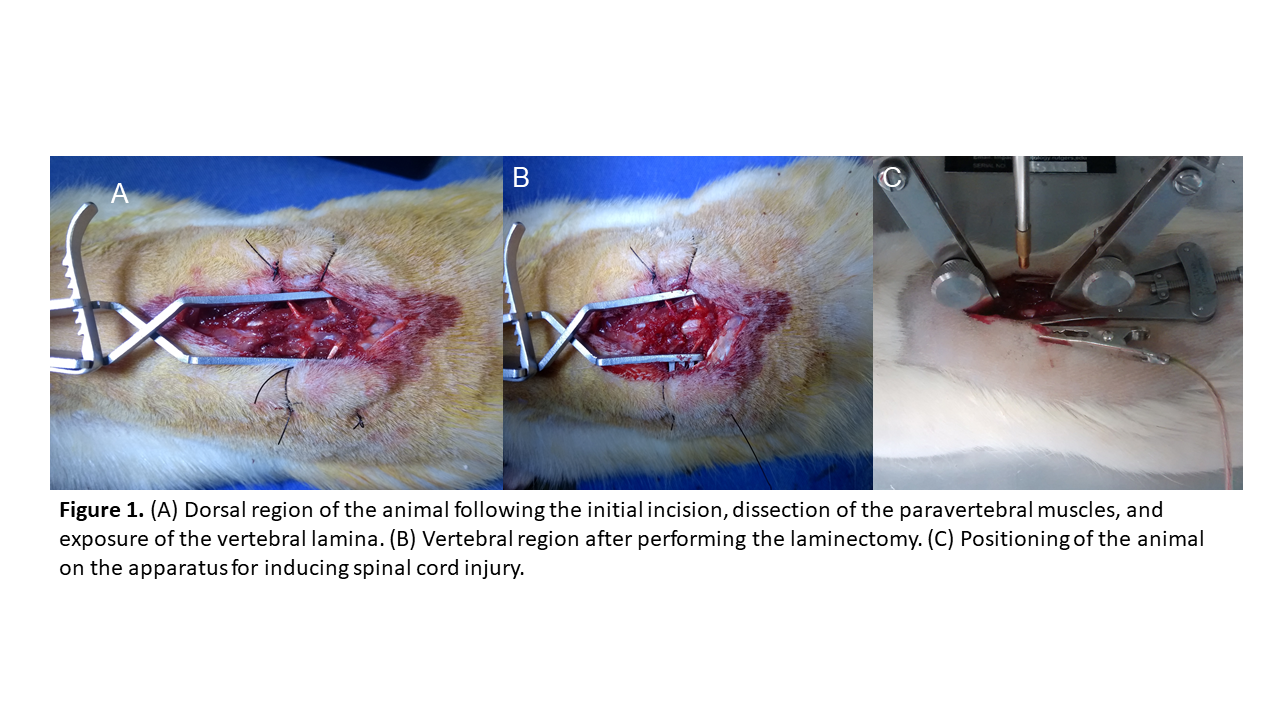

Supplement: Supplementary file 1 [file ijms-25-09043-s001.zip › ijms-3108775-supplementary.tif]
